# Bone Cement Implantation Syndrome: Incidence and Associated Factors in a United States Setting

**DOI:** 10.7759/cureus.31908

**Published:** 2022-11-26

**Authors:** J. Douglas Jaffe, Christopher J Edwards, Rawad Hamzi, Ashish K Khanna, Fredrik Olsen

**Affiliations:** 1 Anesthesiology, Wake Forest School of Medicine, Winston Salem, USA; 2 Anesthesiology and Intensive Care Medicine, Sahlgrenska University Hospital, Gothenburg, SWE

**Keywords:** hip hemiarthroplasty, hip fracture surgery, hypotension during hip hemiarthroplasty, fracture around hip, bone cement implantation syndrome

## Abstract

Background: The incidence of bone cement implantation syndrome (BCIS) following cemented hemiarthroplasty for the treatment of hip fracture in the United States (US) population is not previously described. We sought to describe the incidence and factors associated with BCIS as well as its impact on mortality.

Methods: In this retrospective observational study, electronic health records were examined for all relevant cases and BCIS was identified and graded according to accepted criteria. Demographic data was collected and an adjusted Cox proportional model was performed to determine the impact of severe BCIS on mortality.

Results: Following exclusions for documentation error and misclassification, 69 patients were included in the final analysis. BCIS, regardless of grade, was present in 24 (35%) patients, while severe BCIS (grades 2 and 3) was found in 7 (10%) of patients. Cox proportional hazard estimation adjusted for American Society of Anesthesiologists (ASA) grade, male sex, and age did not show severe BCIS to be independently associated with mortality, hazard ratio (CI) 1.96 (0.22-17.22).

Conclusion: The incidence of BCIS in a selected hip fracture population is comparable in the US to those found in European studies. This study did not establish the occurrence of BCIS with mortality. As cemented hemiarthroplasty is recommended for displaced hip fractures and its use escalates in the US, one can expect an increase in the absolute number of patients experiencing BCIS as well.

## Introduction

An estimated 18% of women and 6% of men worldwide will sustain a hip fracture during their lifetime with rates reported as 789 per 100 000 person-years in women and 240 per 100 000 in men [1]. A progressive increase in the proportion of the population over the age of 65, already the fastest-growing demographic, forebodes a rise in the cumulative annual rate of hip fractures, which currently surpasses 300 000 in the United States (US) [2,3]. Not only are hip fractures associated with significant healthcare costs [4], but they confer a greatly increased mortality rate, with 20-30% of patients dying within the subsequent year following the fracture [5]. For patients who survive, there is often permanent disability, with half never regaining the ability to ambulate without assistance and up to 25% requiring long-term care [6].

Early surgical intervention is the most widely accepted treatment modality to minimize the risk of permanent disability and its attendant comorbidities, particularly for patients with displaced femoral neck fractures [7]. However, surgical intervention for these patients also carries a high in-hospital mortality risk ranging from 3% to 11.4% [8,9]. The most common approach to surgical repair of femoral neck fractures that are partially or completely displaced is bipolar hemiarthroplasty or total hip arthroplasty using either cemented or cementless implants [10]. While the choice to utilize cemented versus cementless implants is largely surgeon-dependent, current practice in the US is shifting toward increasing use of cemented implants. Already the primary method in European countries, cemented implants have the benefit of decreased intraoperative and postoperative fracture risk, as the typically osteoporotic bone has less stress transferred to it with the utilization of cement and does not require additional time for bony ingrowth as is required for cementless [11,12]. Cemented techniques do have drawbacks, however, of typically longer surgical times, increased blood loss, requiring a more meticulous technique, as well as inducing bone cement implantation syndrome (BCIS) [13,14]. The American Association of Orthopaedic Surgeons (AAOS) Clinical Practice Guidelines are in favor of using cemented stems over cementless implants for patients undergoing arthroplasty for femoral neck fractures [15].

Bone cement and its application in orthopedic surgery have been well described [16]. BCIS is a clinical entity defined by hypotension, hypoxemia, and altered mental status usually occurring around the time of cementation of the femoral or acetabular prostheses likely due to embolic fat particles or polymethyl methylmethacrylate (PMMA) polymers, although it can also occur during reaming of the femur, reduction of the joint, and deflation of the tourniquet [16,17].

Previous research has identified the incidence of BCIS to be around 7%, with severe BCIS conferring a 16-fold increase in mortality [18]. Given the ongoing increase in the volume of cemented hip fracture repair in the US [19], as well as the significant clinical impact of BCIS on patient outcomes, the goal is to better characterize BCIS and further identify risk factors for this potentially fatal complication [20]. We sought to examine a cohort of all patients who underwent cemented hip hemiarthroplasty for the repair of femoral neck fracture between 2016 and 2021 at a high-volume US center for orthopedic trauma to measure relative BCIS severity according to Donaldson et al. [17], and risk factors that may influence the predictability for the occurrence of BCIS.

## Materials and methods

A retrospective analysis of anesthetic records was performed after obtaining Institutional Review Board (IRB) approval (IRB 00077618). The requirement for written informed consent was waived, and all applicable EQUATOR/STROBE (Strengthening the Reporting of Observational Studies in Epidemiology) guidelines were referenced and adhered to [21]. All cases of cemented hemiarthroplasty for primary repair of femoral neck fractures occurring between January 1, 2016, and February 5, 2022, occurring at Wake Forest Baptist Medical Center (a tertiary care, university-affiliated hospital) were identified and included in this study. Initial screening for patient inclusion was performed by search of the electronic medical record (EMR) for current procedural terminology (CPT) codes 27236 and 27125 correlating to the performance of a hip hemiarthroplasty. Individual operative notes were then reviewed looking for documentation that specifically indicates a cemented procedure was performed. Subsequently, only cemented cases were included in this analysis.

Patients were excluded from analysis for the following reasons: if the surgical indication was considered non-acute, was secondary to malignancy fracture, was associated with a periprosthetic fracture, and/or was associated with a revision arthroplasty. Additionally, cases that contained incomplete documentation were excluded as well.

For all patients included in this analysis we collected the following demographic, comorbidity, and pharmacologic data: age at surgery, sex, body mass index (BMI), ethnicity, preoperative creatinine, hemoglobin, platelet count, serum sodium, serum potassium, white blood cell (WBC) count, preexisting history of arrhythmia, dementia, malignancy, chronic obstructive pulmonary disease (COPD), congestive heart failure (CHF), myocardial infarction (MI), ischemic heart disease, hypertension (HTN), cerebrovascular accident (CVA), diabetes, renal failure, liver disease/dysfunction, and preoperative use of any of the following medications: beta blocker, diuretic, antiplatelet, organic nitrates, calcium antagonist, ACE (angiotensin-converting enzyme) inhibitor, ARB (angiotensin receptor blocker), insulin, statin, warfarin, or direct oral anticoagulants (DOACs).

The primary outcome of this study was the incidence of BCIS as defined by Donaldson et al. [11] after cemented hemiarthroplasty for primary repair of a femoral neck fracture. Those cases identified as exhibiting BCIS were further subdivided into grade 1 (SaO2 < 94% or fall in systolic blood pressure (SBP) 20-40%), grade 2 (SaO2 < 88% or fall in SBP > 40% or unexplained loss of consciousness), or grade 3 (cardiovascular collapse requiring cardiopulmonary resuscitation (CPR)).

Our secondary endpoints were 30-day mortality stratified by BCIS grade and preexisting comorbidities, as well as the association of preexisting factors on the occurrence of BCIS grades 2 and 3.

**Figure 1:**
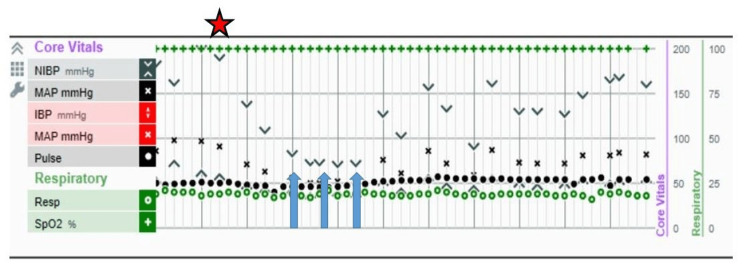
Intraoperative record of a patient with hemodynamic changes immediately following cemented prosthesis implantation (red star) with ensuing hypotension indicated by blood pressure readings below 100 mm Hg (blue arrows).

Identification and grading of BCIS were performed via manual review of the operative and anesthetic records. An example of an anesthetic record demonstrating vital signs changes at the time of documented cementation is presented (Figure 1). The time of cementation was documented electronically in all cases and criteria were assessed during this time period. With regard to diagnosing BCIS, hemodynamic changes had to be temporally associated with one of the following distinctive operative time points: femoral reaming, pulsatile lavage, cementation, prosthesis insertion, and/or reduction of the joint, to be considered a criterion for BCIS.

Statistical analysis

For the evaluation of the primary endpoint, the incidence of BCIS, a descriptive analysis was performed. For the secondary endpoint, that is, the determination of the association between 30-day mortality rates and grade of BCIS, a two-tailed Fischer exact test was performed with BCIS grades 0 and 1 compared to grades 2 and 3 and the proportion of patients deceased within 30-days of surgery. In determining preexisting factors that were associated with the development of BCIS grades 2 and 3, a similar approach is used. The number and selection of preexisting factors to be tested are determined by the size and incidence. The selection of factors is determined by previous literature.

All analyses were done with R version 4.0.5 (R Core Team, Vienna, Austria) with a p-value of < 0.05 considered significant after a two-tailed T-test. Categorical data were represented as counts and percentages, and continuous data as median and interquartile range (IQR). Table 1 reflects the demographic constitution of the patient cohort and includes the incidence of medical diagnoses and medication use among those in the cohort.

## Results

A total of 69 patients were included in the final analysis (Table 1).

**Table 1:** Patient demographics Categorical data displayed as n (%), continuous data as median (IQR) ASA: American Society of Anesthesiologists

Characteristic	N=69
ASA Physical Status (PS) class	
2	3 (4.3%)
3	41 (59%)
4	25 (36%)
Year of surgery	
2017	2 (2.9%)
2018	2 (2.9%)
2019	6 (8.7%)
2020	8 (12%)
2021	43 (62%)
2022	8 (12%)
Age	82 (75, 88)
Ethnicity	
Hispanic or Latino	1 (1.4%)
Not Hispanic or Latino	68 (99%)
Race	
American Indian or Alaska Native	1 (1.4%)
Black or African American	3 (4.3%)
Other	1 (1.4%)
White or Caucasian	64 (93%)
Primary anesthesia type	
General	32 (46%)
Regional	37 (54%)
Gender	
Female	47 (68%)
Male	22 (32%)

Six cases were excluded due to surgery for indications other than primary fracture repair. The final cohort had a median age of 82 years (range 75-88), of which 68% were female. The total incidence of BCIS was 35%, with 30-day mortality for the cohort of 8.7% (Table 2).

**Table 2:** Grade of BCIS and 30-day mortality BCIS: bone cement implantation syndrome

BCIS grade	0 (n=45)	1 (n=17)	2 (n=7)	3 (n=0)
30-day mortality, no	43 (62%)	13 (19%)	7 (10%)	0
30-day mortality, yes	2 (3%)	4 (6%)	0	0

The grade of BCIS was dichotomized to non-severe (grades 0 and 1) and severe (grades 2 and 3). Six patients in the non-severe BCIS group were deceased after 30 days, while there were no deaths in the severe group during the same timeframe. No significant association was found with the rate of 30-day mortality between the patients experiencing severe BCIS compared to those who had no or non-severe BCIS (Table 3).

**Table 3:** Contingency table (2 ⨯ 2) for dichotomized BCIS and 30-day mortality Fisher exact test p=1 BCIS: bone cement implantation syndrome

	BCIS grade 0/1 (n=62)	BCIS grade 2/3 (n=7)
30-day mortality, no	56	7
30-day mortality, yes	6	0

Adjusting for American Society of Anesthesiologists (ASA) Physical Status (PS) class, male gender, and age, there was no statistically significant effect on mortality over time; Hazard ratio for severe BCIS (1.96 [95% CI 0.22-17.22] p=0.5453) compared to patients who did not experience BCIS. The number of confounding factors available for adjustment was limited by the small sample size and an approximate 10% event rate. The selection was based on previous studies and subject matter knowledge (Table 4).

**Table 4:** Variables in the Cox proportional hazard model ASA PS: American Society of Anesthesiologists Physical Status; BCIS: bone cement implantation syndrome; * considered statistically significant (p < 0.05); 95% CI: 95% confidence interval

Variable	Hazard ratio	95% CI	p-Value
ASA PS class	1.77	0.55-5.70	0.3358
Male gender	3.48	1.08-11.3	0.0374*
Age (years)	1.12	1.03-1.22	0.0113*
BCIS grades 2 and 3	1.96	0.22-17.22	0.5453

## Discussion

Our overall rate of BCIS of 35% was comparable to other large registry studies [18,22]. However, our cohort was a single-center experience over a relatively narrow period, and in a selected population demographic. This illustrates that in our highly selected cohort, the rates of BCIS are the same or even higher than in previously reported broad-based populations. As the relative frequency of cemented hemiarthroplasty is on the rise, the absolute number of patients experiencing BCIS will also likely increase. This work sets the stage for the creation of larger BCIS registry datasets across the US, to clearly understand the incidence and outcomes of this important intraoperative complication, which in many cases is preventable and manageable.

Our study’s comparatively small size and retrospective design do present limitations regarding the generalizability of results. At our institution, the majority of hemiarthroplasties are still treated with cementless implants due to surgeon preference, in large part due to concern of BCIS. The timing of cementation was determined electronically, while the other events defined by Donaldson et al. (reaming, lavage, repositioning) were not documented. Surgery and anesthesia notes rarely mentioned an apparent hemodynamic instability in any detail, possibly obscuring relevant episodes from registration; however, automated anesthesia records do show blood pressure data as close to true as possible. Owing to its smaller cohort size, the number of potential confounding factors able to be included in the Cox regression model was very limited. However, we did choose the most important confounders based on previously published data.

One of the strengths of this analysis (that is as a result of the smaller sample size) is that manual data collection and revision were feasible. This led to the correction of several errors in the electronic health record (EHR) data, regarding the registration of comorbidities and medication at the time of surgery. Through manual data extraction and review, we were able to identify the six cases that did not meet selection criteria and were therefore not included in the analysis, as these patients underwent primary surgical repair that was indicated for diagnoses other than a fractured femoral neck.

Recent publications suggest rates of BCIS for hip hemiarthroplasty approaches at a rate of 31% [22] with total incidences of 25-38% [17,18,23] for all cemented hemiarthroplasty procedures for hip fracture treatment. These rates of BCIS seem to be unchanged since the authors’ 2014 study was based on the Donaldson criteria [18].

Of the patient variables analyzed, age showed a very modest positive correlation with BCIS, while male gender showed a significant correlation with BCIS. Our data showed a 3.5-fold increase in BCIS incidence in male patients undergoing cemented hip hemiarthroplasty. While neither age nor gender is modifiable, this finding suggests that, within the context of urgent/emergent surgery, elderly male patients would likely benefit from a more thorough preoperative evaluation specifically assessing for the preexisting cardiopulmonary disease that may indicate an increased risk of morbidity/mortality, were they to develop BCIS. The association might alter anesthetic management as well, by lowering the clinical threshold for use of invasive arterial blood pressure monitoring for male patients. Furthermore, with regard to risk mitigation, the identification of a patient within the highest-risk cohort of older male patients should warrant a discussion between the surgical and anesthetic teams regarding the risks of BCIS compared to the benefits of a cemented prosthesis to aid in the clinical decision-making for operative management of these patients.

## Conclusions

As the trend shifts towards cemented hemiarthroplasty as the predominant surgical management for displaced femoral neck fracture in the elderly and given the current AAOS guidelines and worldwide trends, the number of cases of severe BCIS should be expected to increase. Future research with larger patient cohorts is needed to further characterize the clinical entity of BCIS, standardize its definition to facilitate improved surveillance, and identify additional risk factors, including those that are indeed modifiable, in order to reduce the impact of this potentially fatal complication. Clinical management of BCIS requires a team effort with prompt recognition, transitioning the patient to the supine position to enable initiation of CPR (hemiarthroplasty is frequently performed in the lateral position with supports mounted on the table), and in a prolonged CPR situation, airway management, and continued resuscitation. All operating room staff involved in cemented procedures in general, and hemiarthroplasty of the hip in particular, should be trained to surveil for and be prepared to manage the sequelae of BCIS.
